# Frequent and biased odorant receptor (OR) re-selection in an olfactory placode-derived cell line

**DOI:** 10.1371/journal.pone.0204604

**Published:** 2018-09-26

**Authors:** J. C. Noble, Diane Meredith, Robert P. Lane

**Affiliations:** Department of Molecular Biology and Biochemistry, Wesleyan University, Middletown, Connecticut, United States of America; Duke University, UNITED STATES

## Abstract

We previously characterized a clonal olfactory placode-derived cell line (OP6) as a model system for studying odorant receptor (OR) choice, where individual OP6 cells, similar to olfactory sensory neurons *in vivo*, transcribe one allele (“monoallelic”) of one OR gene (“monogenic”). The OP6 cell line provides a unique opportunity to investigate intrinsic properties of OR regulation that cannot easily be investigated *in vivo*. First, whereas OR-expressing cells *in vivo* are post-mitotic, OP6 cells are immortalized, raising interesting questions about the stability of epigenetic states associated with OR selection/silencing as OP6 cells progress through the cell cycle. Second, OP6 cells have been isolated away from extrinsic developmental cues, and therefore, any long-term OR selection biases are likely to arise from intrinsic epigenetic states that persist in the absence of developmental context. In this study, we investigated OR re-selection frequency and selection biases within clonal OP6 cell populations. We found no evidence of OR stability through the cell cycle: our results were most consistent with OR re-selection events transpiring at least once per cell division, suggesting that chromatin states associated with OR selection in this system might not be maintained in the subsequent generation. In contrast, we found strong evidence for OR selection biases maintained over prolonged culturing across a diverse set of OP6 cell lineages, suggesting the persistence of intrinsic epigenetic states that advantage some OR loci over others. Together, our data suggest that in the absence of instructive cues, intrinsic epigenetic states influencing OR eligibility, but not those determining OR choice, might persist through the cell cycle.

## Introduction

The sensory neurons of the mammalian olfactory system are specialized for odorant binding function as a consequence of expressing only one type of olfactory receptor (OR) protein in each cell [1–4]. Mutually exclusive OR gene expression occurs despite the very large number of OR genes encoded in a typical mammalian genome; in mouse, there are ~1,400 OR genes organized in numerous clusters of various sizes distributed on nearly every chromosome [5, 6]. While significant progress has been made in recent years, it remains unclear how each olfactory sensory neuron (OSN) comes to express one parental allele (“monoallelic”) of one only one OR gene (“monogenic”), while keeping the remaining enormous repertoire of OR genes silenced, including neighboring OR genes clustered in the immediate vicinity of the chosen OR.

Recent evidence points to a stochastic and iterative process, whereby subsets of OR genes are specified as “eligible” based on the developmental niche in which the OSN arises [7, 8], an apparently stochastic selection is made among this eligible OR subset, and this choice is stabilized by commitment mechanisms that include feedback loops and chromatin modifications [9, 10]. In mouse, these stepwise processes–*specification*, *selection*, *and commitment*–occur within the olfactory epithelium (OE) along a basal to apical developmental gradient [11–13]. Among the most basal cells are stem cells that appear to be largely unspecified with respect to OR selection [14]. The most mature OSNs are located in the apical-most layer, committed to express a single OR protein on the surface of ciliated dendrites that extend into the nasal cavity [15–17]. Between the innermost and outermost layers of the OE are developing, post-mitotic OSNs at various stages of maturation, as characterized by their gene expression profiles [18–20]. The range of OR options “eligible” for selection in a differentiating OSN is influenced by its environment, both temporally and spatially [8, 21, 22]. During this early “specification” stage, some immature neurons express low levels of multiple OR genes at once [23, 24]. Nevertheless, by the time the OSN has matured fully, only one OR allele is robustly transcribed, with the remaining ORs silenced within heterochromatic compartments of the nucleus [25, 26]. This last “commitment” stage involves a feedback loop in which the presence of a properly-folded OR protein on the cell surface triggers a signal cascade that prevents any additional OR activations in that cell [9]. In the absence of a bonafide OR protein (e.g., in the event an OR pseudogene has been haphazardly selected), this feedback loop fails to be executed, thus permitting the cell to select another OR prior to commitment (OR “switching”) [27, 28]. OR “switching” only rarely occurs *in vivo*, presumably because pseudogenes are relatively unlikely to be initially selected and because the kinetics of the feedback pathway are likely much faster than that of the reselection pathway.

We have used an immortalized cell line (OP6) derived from a single olfactory placode (OP) cell isolated from the ~E10 developing mouse OE as a model system for investigating mechanisms of OR regulation [29]. We have previously characterized this model system as obeying the “one cell, one OR” rule–that is, each OP6 cell in the culture appears to regulate OR expression both monoallelically and monogenically [30]. OP6 cells have been characterized as “immature” OSNs by developmental gene expression profiling [29–32], and exhibit additional features consistent with a premature state along the lineage, including reduced chromocenter aggregation and low OR expression levels [25, 30]. When further differentiated using retinoic acid, OP6 cells develop into characteristic bipolar morphology, as well as exhibit chromocenter condensation and developmental gene expression profiles characteristic of a more mature state, substantiating the OP6 founder as an immature OSN [25, 29–31]. One interesting feature of the OP6 line stems from its immortalization–while expressing only one OR per cell, this choice is not stable during culturing, suggesting that dividing OP6 cells are unable (or less able) to “commit” to an OR [30, 31, 33]. Therefore, the OP6 cell line represents an opportunity to decouple the “OR specification”/“OR selection” process from the “OR commitment” process, a scenario that is difficult to achieve *in vivo*. Moreover, this model system permits investigation of epigenetic properties of OR expression and silencing in the context of the cell cycle.

In this study, we investigate OR re-selection tendencies in OP6 cultures with two goals in mind. First, we were interested to know whether there persists any “memory” of OR selection through the cell cycle, as opposed to a systematic reprogramming of OR choice occurring with each cell division. Second, we were interested to know whether OR re-selection is stochastic or biased in nature, the latter possibly consistent with an intrinsic and heritable epigenetic state that provides competitive selection advantages. We show that OR re-selection in OP6 cultures is frequent, occurring at least once per cell cycle, suggesting that any chromatin remodeling associated with initial OR choice in this system was not maintained through the cell cycle. In contrast, we found strong evidence for persistent OR selection biases, suggesting the presence of intrinsic epigenetic states influencing the competitive balance among OR gene loci.

## Materials and methods

### OP6 cell cultures

The OP6 cell line was cultured at 33 °C in *Dulbecco's Modified Eagle's Medium* (DMEM, Life Technologies) supplemented with 10% fetal bovine serum (Gibco), as described previously [29]. For RNA FISH, cells were seeded on 22cm^2^ coverslips coated with 0.1% gelatin (Sigma) in a 6 well plate at about 50% confluency and expanded for one day until near confluency. For colony RNA FISH, cells were seeded at ~2,000 cells per slide and grown for 7–8 days (50 cell colonies) or at ~10,000 cells per slide (4 cell colonies), and grown for 2–4 days.

### RNA FISH

Long intron probes for some RNA FISH experiments were synthesized using *Long Range polymerase* (Qiagen) with sequence-specific primers (see S1 Table) and incorporation of *DIG-16-dUTP* (Sigma) into PCR products. We utilized intron probes for OR RNA FISH for three reasons: (i) we can design longer intron than exon probes (enhanced sensitivity); (ii) for genes (like ORs) expressed at low levels, unprocessed RNAs at the native locus are more spatially concentrated than processed RNAs in the cytoplasm (enhanced sensitivity); (iii) the one-spot (monoallelic) nuclear signal is an important validation of an OR signal (enhanced specificity). For most RNA FISH experiments, the long-intron PCR products (PCR primer sequences are provided in S1 Table) were cloned into *pCRII-TOPO* vector (Invitrogen); prepared plasmids were linearized prior to *in vitro* transcription using *SP6* or *T7* polymerases (Roche) for production of sense- or antisense-specific probes with incorporation of *DIG-16-dUTP* or *Biotin-16-dUTP* (Sigma). 100 ng of labeled probe was combined with 5 μg *Cot1-DNA* (Invitrogen) and 10mg salmon sperm DNA (Sigma) in a 2 ×*SSC*, 10% dextran sulfate solution, and heat denatured. For two-color RNA FISH colony experiments, 20ug of E. coli tRNA (Sigma) and 50ug of BSA (Sigma) were added to reduce background. Cells were permeabilized with 0.5% Triton-X in *CSK* buffer, fixed with 4% paraformaldehyde in *PBS*, and dehydrated in a 70%–80%–95%–100% ethanol series. Probe and cells were incubated overnight at 37°C in a humidified chamber. Following washes (maximum stringency = 50% formamide, 0.5 ×*SSC* at 37°C), samples were blocked for subsequent antibody incubations (4% *BSA*, 4 ×*SSC*, 0.2% *Tween-20*). DIG signals were visualized using sheep anti-DIG *FITC* (11207741910, Roche) and donkey anti-sheep *FITC* (sc-2476, Santa Cruz Biotech) antibodies, at a 1:100 dilution in 1% *BSA*, 4 ×*SSC*, 0.2% *Tween-20*. Biotin signals were visualized using *avidin-DCS rhodamine* (A2012, Vector Labs), followed by *biotinylated anti-avidin* antibody (Ab73235, Abcam) plus an additional incubation with the *avidin-DCS rhodamine*, each used at 1:100 dilution in 1% *BSA*, 4 ×*SSC*, 0.2% *Tween-20*. For OR re-selection assays, we used 6 long-intronic DNA probes against 40 small colonies for each probe (240 total colonies; 975 total cells were screened) and 6 larger colonies each (36 total colonies; 2,497 total cells were screened). In addition, we conducted two-color RNA FISH experiments using the *Olfr920* (labeled with DIG) and *Olfr57* (labeled with biotin) probes in order to investigate whether small colonies are able to activate more than one OR gene. For measuring expression frequencies in a well-defined lineage, we used sense/antisense RNA probes against 9 OR genes on 28 total cultures (2–5 replica cultures per probe). Images were acquired using a *Deltavision RT imaging system* (Applied Precision) adapted to an Olympus (*IX71*) microscope equipped with *XYZ* motorized stage. Each image was sectioned with 0.5 μm intervals to ensure complete coverage of the nucleus. *ImageJ* (Fiji) was used for analysis of positive cells.

### Method considerations for OR profiling

We considered various methodologies for profiling OR representation in OP6 cell populations. We estimate that OR mRNA yield from a positive OP6 cell is roughly in the 10–100 template range based on relative expression levels of abundant ORs from previous qPCR experiments. For example, if we assume that frequently represented OR genes are expressed in ~1–2% of OP6 cells (as observed in RNA FISH experiments herein, see text), then observed *actin*:*OR* cDNA ratios in full OP6 cell populations would be ~50-100-fold greater than the *actin*:*OR* cDNA ratio in single positive cells. The observed *actin*:*OR* ratio for a set of commonly expressed OR genes (e.g., *Olfr920*, *Olfr544*, and *Olfr57*) averaged ~6,500:1 (not shown), suggesting ~65-130-fold actin excess in single OR-positive cells. We approximate the *actin* transcript abundance at ~10^3^−10^4^ molecules per cell [34–36], and therefore estimate the OR transcript abundance to be between ~8 mRNAs (assuming ~10^3^ actin mRNAs at ~130-fold excess relative to OR mRNA) and ~150 mRNAs (assuming ~10^4^ actin mRNAs at ~65-fold excess relative to OR mRNA). These estimates are consistent with OR template numbers for relatively low-abundance genes in typical cells.

With this low OR transcript abundance in mind, we decided against using RNA-seq for three reasons: (1) Assuming a positive OP6 cell contains at most ~100 OR transcripts, and estimating ~10^5^ total RNA transcripts per cell [37, 38], we reasoned that the putative median OR in the population (expressed in ~1/1,000 OP6 cells) might be <1 transcript per million transcripts from an OP6 population (or, <1 FPKM). This is commonly the noise cutoff threshold used in *RNA-seq* to account for false-positives in alignment or other technical limitations [39, 40]. (2) In order to obtain a reliable number of sequence reads per OR (e.g., >100 hits per gene), we would need to sequence to a depth >100 million reads per sample, which was not financially feasible when considering our goal of characterizing numerous OP6 populations within several well-defined lineages. (3) The cDNA preparation protocols commonly utilized prior to sequencing involve competitive enrichment (e.g., polyA isolation, PCR-based amplifications), which potentially introduces methodological biases that are particularly skewed against low-copy number transcripts [41, 42]. Obviously, we did not want potential methodological biases to obscure our interpretation of apparent OR expression biases. To achieve the desired sensitivity while mitigating potential amplification bias, we developed a nested PCR strategy detailed in the following section.

### Nested PCR

We optimized cDNA preparation and nested PCR protocols to identify a condition for each OR gene tested that *(a)* reliably and reproducibly gave robust products on gDNA diluted to <100 templates, and *(b)* reliably and reproducibly did not generate products in no-RT controls for various RNA preparations from OP6 populations. We were able to develop nested PCR assays that satisfy the above criteria for 21 OR genes. Each OR gene was investigated in cell populations across various OP6 cell lineages. Approximately 5x10^6^ OP6 cells per culture were harvested and RNA was extracted using *Trizol* (Thermo Fisher/Life Technologies). Approximately 5 μg of RNA was treated with DNase (Thermo Fisher/Ambion) and further purified using the *RNeasy* Mini Kit (Qiagen). Approximately 500 ng of resulting RNA was subjected to first-strand cDNA synthesis by *SmartScribe* reverse transcriptase (Clontech), followed by PCR using multiplexed OR primer pairs for 20 cycles. A second nested PCR reaction was conducted for the 21 OR genes (individual reactions, not multiplexed) with cycle numbers optimized to report 50–100 OR templates from known gDNA quantities, while maintaining cleanly negative results in no-RT controls from various OP6 cell populations. All PCR primer sequences are provided in S1 Table.

The <100 template sensitivity threshold was chosen because we estimate that this is approximately the OR template yield from one positive OP6 cell (see above). In our analyses of various OP6 cell populations, we input unamplified cDNA corresponding to the equivalent of ~400 and ~2,000 OP6 cells, thus reporting ORs that we estimate are expressed in >1/400 (>0.25%) and >1/2000 (>0.05%) cells, respectively. Therefore, these two sensitivity thresholds should permit surveying the middle portions and upper half of a normal distribution that models OR frequencies for the null hypothesis in which all ORs are represented by a completely stochastic selection process.

## Results

We have previously shown that only one OR allele (“monoallelic”) of one OR gene (“monogenic”) is expressed at detectable levels in individual OP6 cells, and that many OR genes are expressed in entire OP6 cultures at various passages [30, 31, 33]. Given that OP6 cultures are clonally derived [29], these observations indicate that OR re-selection has occurred–if OR choice was stable, we would predict that OP6 cultures would be ~homogeneous with respect to OR expression. Therefore, these observations raise two interesting questions about the epigenetics of OR choice in this system: (1) Does the cell remember its choice through the cell cycle (i.e., as would be predicted if stable epigenetic states are established with the initiation of OR expression/silencing)? (2) Is OR choice persistently biased (e.g., as a form of epigenetic memory lingering from the developmental niche from which the OP6 founder cell was isolated or by intrinsic epigenetic states that vary with genome context)? We address the former question with RNA FISH using a sample of OR probes on small OP6 colonies to investigate whether OR expression persists in progeny cells, and we address the latter question using RNA FISH and nested PCR methods to interrogate OR expression profiles over a defined lineage of OP6 cultures.

### OP6 cells frequently re-select OR genes during culturing

**Fig 1:**
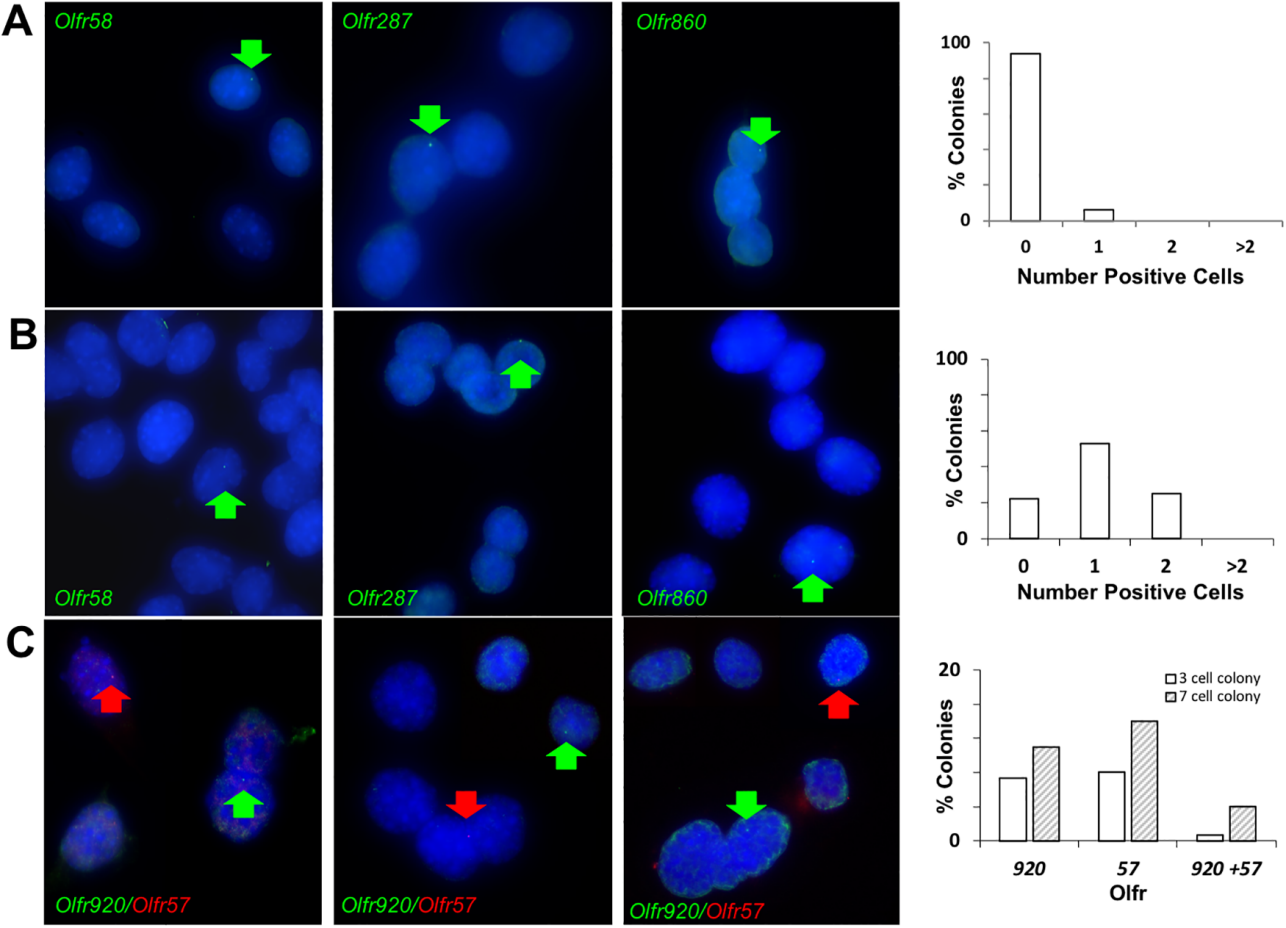
RNA FISH on OP6 colonies to investigate OR re-selection frequency. RNA FISH was conducted on 4-cell (Panel A) and 50-cell (Panel B) OP6 cell colonies using six different OR probes (*Olfr58*, *287*, *860*, *378*, *69*, *and 868*). Representative images are shown for 4-cell (panel ***A***) and 50-cell (panel ***B***) colonies for *Olfr58*, *287*, and *860*, each exhibiting one positive cell within the colony (green arrows). The entire set of RNA FISH images is shown in S1 Fig. A total of 240 of the 4-cell colonies (40 colonies per probe) and 36 of the 50-cell colonies (6 colonies per probe) were analyzed. The percentage of colonies exhibiting 0, 1, 2, or >2 positive cells per colony is shown in the histograms (right panels). **C**. Two-color RNA FISH using probes against *Olfr920* (green) and *Olfr57* (red) illustrating a 3-cell colony (left panel) and two 7-cell colonies (right panels) containing a positive cell for each probe. A total of 150 of the ~3-cell colonies (455 total cells) and 100 of the ~7-cell colonies (688 total cells) were analyzed. The histogram (right panel) shows percentages of ~3-cell colonies (open bars) and ~7-cell colonies (hashed bars) containing a positive cell for *Olfr920* or *Olfr57*, as well as those containing a positive cell for both ORs.

We isolated single OP6 cells and grew out colonies in order to investigate whether OR expression persists through the cell cycle (Fig 1). We screened a large number of 4-cell colonies using RNA FISH to identify rare colonies containing a positive cell. For this experiment, we designed probes against a set of 6 OR genes that we knew from previous RNA FISH experiments would be expressed at high frequency in random OP6 cell populations: *Olfr287*, *Olfr58*, *Olfr860*, *Olfr378*, *Olfr69*, and *Olfr868*. We observed that all of these probes exhibited positive RNA FISH signals in 1–2.5% of sampled cell populations (not shown).

The percentage of positive cells within clones approximated the percentage of positive cells observed in large OP6 populations. For example, the *Olfr860* probe tends to exhibit a robust RNA FISH signal in ~2% of cells in large OP6 cultures, and we observed ~1.8% of the cells within the 4-cell colonies positive for this probe. As anticipated, most colonies (~94%) were negative for a given OR probe. Of the 15 colonies that were positive for a particular OR probe, none of these colonies contained more than one positive cell per clone (Fig 1A). Therefore, we find no evidence to suggest that a given OR gene remains active for more than a single generation.

A possible caveat to this conclusion is the false-negative rate for a given RNA FISH probe, which could lead to an underestimation of double-positive colonies. We addressed this caveat in two ways. First, we generally assessed false-negative rates for the RNA FISH method in our hands using a panel of non-OR probes known to be expressed in all OP6 cells (e.g., *Actin*). From experiment to experiment, various non-OR probes exhibited a false-negative rate ranging between ~5–35%, with the maximum rates typically observed for small (<500-bp) intron probes. We note that we have designed RNA FISH probes against introns for detection of nascent transcripts in order to enhance sensitivity for the purpose of detecting very low expression levels (see Methods). Detection of nascent transcripts using this method for all OR genes tested produced signal:noise intensities as robust as any housekeeping gene probe. Moreover as noted previously, several antisense (but not sense) OR probes report a positive incidence within OP6 cell populations that exceed 1% (i.e., >10-fold higher frequency than the expected median frequency assuming a repertoire of ~1,000 OR genes), results that seem to argue against widespread under-detection. Therefore, we have no reason to suspect that the false-negative rate for various OR probes falls beyond this ~5–35% range observed for various non-OR probes. Nevertheless, even assuming a false-negative rate as high as 50% (e.g., in such a scenario, the true positive incidence of *Olfr860* might be 3%, not 2% as observed), we can dismiss the hypothesis that OR expression is typically retained or remembered through the cell cycle, since the probability (*p*) of observing *n* divisions involving a positive cell without observing signal in both progeny cells is *(0*.*5)*^*n*^. Our sample size was 15 positive colonies, each of which contained two cell divisions (from single cell to 4-cell colony), therefore, *n* = ~60 cell division opportunities. Therefore, it is very unlikely in such a large sample of opportunities that we would never observe a double-positive colony if the same OR commonly remained active from one generation to the next (*p<~10*^*−15*^).

A second way we addressed this possible “false-negative” caveat was to grow much larger colonies (>50 cells) in order to provide greater opportunity for these colonies to reveal sustained OR selections during a larger clonal expansion (Fig 1B). As before, the overall percentages of positive cells across these larger cell colonies (average colony size = ~70 cells) very closely approximated frequencies generally observed in large OP6 populations. For example, as noted previously, the *Olfr860* probe tended to exhibit a robust RNA FISH signal in ~2% of cells in large OP6 cultures and we found the same ~2% (8 of 458 cells) positive rate across multiple colonies. Whereas 4-cell colonies exhibiting a positive signal for a given OR probe were rare in the previous experiment (~6% of 4-cell colonies were positive for a given OR), identifying a larger colony containing a positive signal for a given OR probe was routine (e.g., all six of the larger cell colonies examined were positive for the *Olfr58* probe in at least one of the cells per clone). Overall across a panel of six OR probes, we found that ~78% of the colonies tested positive (n = 28 positive colonies). Nevertheless, despite the larger size of these colonies (~70-cell versus 4-cell), in which each colony had ~35 (versus 2) terminal mitotic divisions as opportunities to reveal OR stability in progeny cells, we still observed that most of the positive colonies (19/28 = ~68%) contained only one positive cell per colony per probe (Fig 1B). The other 9 positive colonies contained two positive cells, however, in 8 of these cases, the two positive signals were present in cells that were distantly separated from each other within the colony. Therefore, we presume that the presence of a second positive within a given colony was probably an independent as opposed to an inherited OR choice made at different points in time during colony expansion. We note that the null hypothesis (e.g., a Poisson distribution) would predict that some ~70-cell colonies would indeed contain two cells independently by chance alone. For example, a 2% positive incidence for *Olfr860* would predict an average of ~1.4 positive cells per ~70-cell colony.

The absence of sustained OR expression through cell division events does not necessarily mean that OR re-selection has occurred; for example, it is possible that progression through the cell cycle has destabilized OR expression (the OR no longer is expressed) without activating a new OR gene. *A priori*, an argument against this hypothesis is that OR representation (i.e., the fraction of OP6 cells expressing any given OR gene) is not decreasing over time. That is, OR de-activation and re-selection appear to have balanced probabilities, given that the percentage of cells expressing various OR genes has remained ~constant over prolonged culturing of the cell line. To demonstrate that re-selection has occurred with every cell division, we would need to show that all cells of a typical colony were positive for a different OR gene, which would require the development of a large, multi-colored pool of RNA FISH probes (e.g., perhaps dozens of pooled OR probes) that report a large fraction of OR-expressing cells; however, we have not yet been able to scale pools of RNA FISH probes beyond 3–4 probes per experiment without observing increased false-negative rates.

So instead, we sought to document explicit selection of two different OR genes in the context of a small clonal expansion as evidence that OR re-selection is able to occur on that time scale and with a frequency consistent with observations made in whole populations. We conducted two-color RNA FISH experiments using a pair of robustly-expressed OR genes (*Olfr920*, *Olfr57*) on 150 small colonies each consisting of 2–4 cells (455 total cells, average colony size = 3.0 cells) and 100 slightly larger colonies each consisting of 6–8 cells (688 total cells, average size = 6.9 cells). The overall percentages of positive cells (*Olfr920* = ~2.5%, *Olfr57* = ~2.9%) were consistent with control experiments on whole populations using non-pooled probes, so no interference was evident as a consequence of pooling the two probes; we also observed similar background levels with sense-strand control pools (~0.6%) as had been observed in parallel non-pooled experiments. Assuming independent probabilities for the selection of these two ORs, we would expect to observe ~0.6 (of 150) 3-cell colonies and ~2.6 (of 100) 7-cell colonies containing a positive cell for both probes. We observed one 3-cell colony and four 7-cell colonies expressing both ORs (Fig 1C), an observed incidence that is not significantly different than the expected incidence. These results confirm that small OP6 colonies are able to re-select a different OR within the time frame of <2 cell cycle events, and suggest that the incidence of OR re-selection within small colonies is consistent with OR representation in larger populations. Together with the observation that OR representation does not appear to decline over prolonged passaging (and thus, OR deactivations are apparently balanced with activations), the most parsimonious interpretation would seem to be that a new OR is activated after each cell division.

Together, our RNA FISH data on OP6 colonies indicated that OR expression does not generally persist through cell division, with apparent OR re-selection occurring as frequently as each cell cycle. Such an outcome is not consistent with the establishment of a stable epigenetic state that determines or strongly influences OR expression/silencing in the subsequent generation. Rather, these results suggest that any chromatin states established with OR selection in this system are neutralized by chromatin reorganization during the cell cycle.

### Investigation of OR re-selection biases in the expansion of the OP6 cell line

From the above data, we concluded that OP6 cells re-select the expressed OR gene at least as frequently as once per cell division. We were next interested to know whether OR re-selection in the OP6 cell line is biased or stochastic in nature. If OP6 cells are biased with respect to OR re-selection, they might select from a narrow range of eligible OR genes–e.g., as a consequence of intrinsic (i.e., lineage-based) specification that is maintained as a heritable trait of the cell line. This hypothesis predicts that the same subset of OR genes will be represented in OP6 cell populations, irrespective of passage and lineage relationships, and that many/most OR genes will not be expressed because they are “ineligible” in the specified state. At the other extreme (null hypothesis), in the absence of any heritable specification, OP6 cells might randomly select an OR gene during a re-selection event where the selection of one OR is ~equally likely as any other of the ~1,000 OR genes encoded in the mouse genome. This null hypothesis predicts that large OP6 cell populations (e.g., >10^6^ cells) will have every OR represented, with a ~monomodal distribution centered at ~0.1% expression frequency (i.e., the median OR would be expressed in approximately 1 cell per thousand-cell population), however, such randomness would predict varying high- and low-expressers from population to population. Finally, there are numerous possible scenarios between these two extreme hypotheses; e.g., “drift” over time to increasingly more or less random-looking representation, or “modulation” over time from one biased OR subset to another. We sought to distinguish among these possible scenarios, in order to gain insights into intrinsic epigenetic properties influencing OR selection probabilities in the absence of *in vivo* developmental influence.

For this study, we sought to identify a methodology that would permit deep investigation of OR representation in OP6 cell populations; e.g., that would confidently report OR transcript presence for an OR gene expressed in merely ~1 in 1,000 OP6 cells in the population (the theoretical median of an unbiased representation). We decided against *RNA-seq* methodology for reasons detailed in the Methods (see “Method Considerations for OR Profiling”). Instead, we turned to two independent methods to investigate OR selection trends in OP6 cell populations: RNA FISH and a nested PCR strategy. RNA FISH is advantageous because it provides resolution at the level of single cells (e.g., to directly measure expression frequency with populations). This layer of information cannot be parsed from global cDNA measurements (e.g., *by RNA-seq*) because overall transcript levels are a function of both the frequency of positive cells and the level of expression per cell. However, the RNA FISH method is prone to false-positive signals, making measurements of rarely expressed ORs problematic. In our hands, negative control RNA FISH probes (e.g., sense probes, or antisense probes for genes presumed not to be expressed in a particular cell type) nevertheless produce a positive signal in ~1–2 nuclei per 300 cells (~0.6% average; as observed in subsequent RNA FISH experiments), and therefore, the “noise” level is greater in incidence to the signal (putative median OR expresser, ~0.1% incidence) we would like to be able to measure. This false-positive issue is even more problematic in the context of OR genes, which express monoallelically, and therefore, it is more difficult to distinguish a true positive from a false positive, since there is no confirming second signal per nucleus. Therefore, although RNA FISH is desirable in that it provides direct read-out of the expression profile (percentage of positive cells), it is reliable for only surveying the most abundantly represented OR genes. The nested PCR strategy we developed (see Methods) reliably delivered the necessary sensitivity to evaluate the expression status of very low-expressing OR genes (i.e., the putative median OR expresser, ~0.1% incidence) without competitive template skewing that can contribute to misrepresentation of OR expression levels. Together, the combination of RNA FISH and nested PCR therefore provided two independent dimensions of information, with the former useful for direct measurement of OR selection probabilities (but for more robustly expressed ORs whose expression frequency is well above anticipated RNA FISH noise levels) and the latter useful for achieving sensitivity levels that permit deeper interrogation of OR representation (see subsequent section).

#### Nested PCR amplification of unamplified OR cDNA

We developed nested PCR assays for 21 OR genes that span a range of expected expression levels based on previous qPCR and RNA FISH experiments (see Methods). We note that we aimed to simulate a random sampling of OR genes for this study, in order to capture as broad a dynamic expression range as possible, and therefore, we only included a single OR gene (*Olfr287*) that we had previously identified as a “robust expresser” in a typical OP6 cell population, as reported in Fig 1.

**Fig 2:**
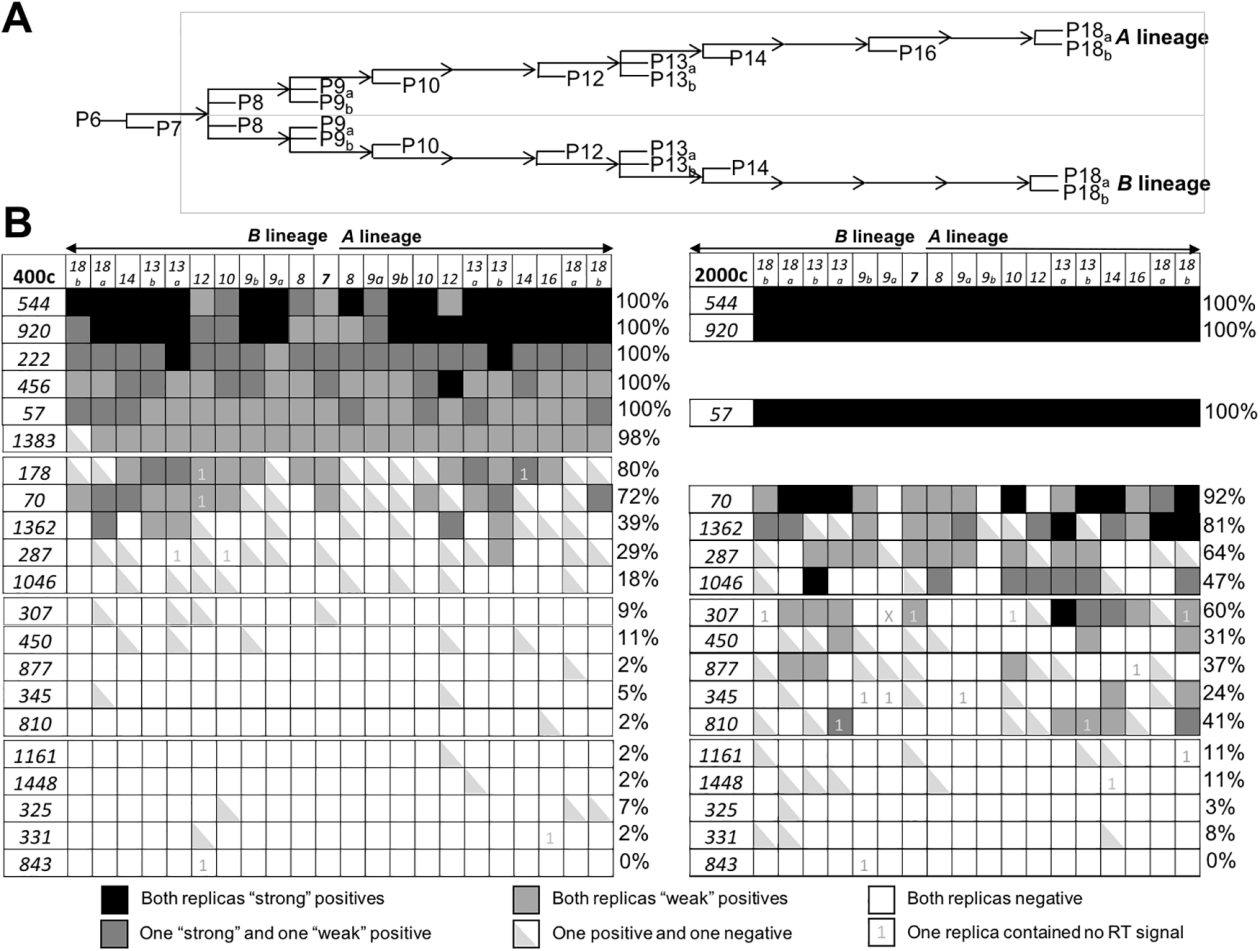
OR expression analysis in 22 OP6 populations across two diverging lineages. **A**. Lineage tree starting from a common P6 cell population. At indicated passages, cell populations were split and grown to confluency for subsequent passaging, with one of the split populations retained for cDNA analysis at P7, P8, P10, P12, P14, and P16 (*A* lineage only), or two sibling populations retained at P9, P13, and P18. **B**. Tables illustrating nested PCR results for 21 OR genes (*Olfr*, left column) across 22 harvested cultures using cDNA amounts equivalent to ~400 OP6 cells (left panel) and across 18 of these cultures for 17 of these ORs using cDNA amounts equivalent to ~2000 OP6 cells (right panel). Each nested reaction was conducted in duplicate from the original unamplified (first-strand) cDNA. Shading within each cell summarizes PCR results for a given OR (rows) on a given cell population (columns); the shading legend is provided below the table. Duplicate robust positive bands (>100 templates) are indicated by black shading, duplicate reactions with one robust (>100 templates) and one faint (<100 templates) positive bands are shaded dark gray, and duplicate faint positive bands (<100 templates) are shaded light gray. In some samples, the OR seems to be very near detection thresholds, as evident by both a faint positive band and a negative in the duplicate experiments (half-shaded cells). Empty cells indicate duplicate negatives for that OR in a given sample. Representative gel images for one population (P13a, Lineage-*A*) are provided in S2 Fig. All scored samples exhibit clean negatives in no-RT controls. Several experiments contained faint products in no-RT controls for one of the two replicas (indicated by “1”), so these specific samples are scored based on a single experiment; one experiment (*Olfr307* in *P9b* of *Lineage-B)* was not scored due to no-RT bands in both replicas (*X*). For scoring purposes, reproducible positives (irrespective of the intensity of the product) were valued as one occurrence, and ambiguous positives (half-shaded) were valued as 0.5 occurrences. The percentage of scored samples with above-threshold PCR products for each OR at each input level is indicated in the leftmost column in each table.

We derived two OP6 lineages (*Lineage-A*, *Lineage-B*) from a common P6 frozen cell stock, harvesting at P7, as well as at various points along the two lineages (see lineage tree in Fig 2A). At P9, P13, and P18 along both lineages, we harvested two parallel “sibling” populations. We conducted duplicate nested PCR reactions on unamplified cDNA isolated from the P7 population, 11 populations along the A lineage, and 10 populations along the B lineage (22 total populations). We tested all 21 OR genes across all 22 populations at the more sensitive input level (400c). We additionally tested 17 (of 21) ORs on 18 (of 22) populations at the higher input level (2000c). Representative gel images are provided in Supplementary Data (S2 Fig) for the P13/*Lineage*-A population, illustrating 100-template gDNA sensitivity, absence of signal in no-RT controls, and presence/absence of signal in cDNA at the 400- and 2000-cell input levels for replica experiments conducted on all 21 OR genes. A summary of results for the entire experiment is shown in Fig 2B.

#### ORs with persistent robust levels of expression

The most striking observation was that six ORs (*Olfr920*, *544*, *57*, *222*, *456*, *and 1383*) were reproducibly above-threshold in all 22 cultures at the ~400c input level. If the 400c input level reports an OR expressed in >1/400 cells (>0.25%) as estimated, any OR reporting a positive product would be designated as well “above average” (>>0.1%) in a distribution modeling the null hypothesis in which random OR selection occurs from any of the ~1,000 OR repertoire. In a normal distribution modeling this null hypothesis, the probability that any random OR gene would be “above average” in 22 of 22 samples is (0.5)^22^ = ~10^−7^ (1 occurrence in ~10 million opportunities). We observed six such occurrences in 21 opportunities, an extremely unlikely outcome that is not consistent with this null hypothesis. Moreover, we note that five of these ORs generally exhibited robust positive PCR bands (i.e., a product that exceeded the 100-template gDNA control in the experiment; denoted by darker shading in Fig 2B): *Olfr544* (~80% of the cultures), *Olfr920* (~77% of the cultures), *Olfr222* (~52% of the cultures), *Olfr456* (~20% of the cultures), and *Olfr57* (~16% of the cultures) were routinely more robust products than the 100-template gDNA control. Only 3 other ORs exhibited rare robust PCR products at this input level (*Olfr70*, *Olfr178*, and *Olfr1362* in ~20%, ~14%, and ~11% of samples, respectively); the remaining 13 ORs never produced a robust PCR product (i.e., >100-template gDNA control) in any of the samples.

We also conducted nested PCR on a third lineage (*Lineage-C*), grown from a different stock of frozen P6 OP6 cells, harvesting 6 populations (at P8, two at P9, P12, and two at P13). We found that these six ORs were consistently positive in each of these cultures at the 400c input level as well (Suppl. Data, S3 Fig), including consistently robust PCR products (denoted by darker shading in figure) for *Olfr544* (100% of cultures), *Olfr920* (100% of cultures), *Olfr456* (100% of cultures), and *Olfr1383* (~83% of cultures), whereas only one of the other 102 samples tested with other OR genes gave a robust PCR product (P12 culture for *Olfr877*). Therefore, these six OR genes were significant outliers in terms of their consistency of appearance and robust signal strength across a large and diverse set of OP6 populations spanning three different lineages.

#### ORs with persistent below-threshold expression

At the opposite end of the spectrum, 11 of the 21 ORs tested by nested PCR (*Olfr1046*, *307*, *450*, *877*, *345*, *810*, *1161*, *1448*, *325*, *331*, *and 843*) in the lineage study did not produce a single, reproducible positive PCR band in any of the 22 populations at the 400c input level (Fig 2B). While some of these ORs produce rare reproducible positive PCR bands at the higher 2000c input level, 5 ORs (*Olfr1161*, *1148*, *325*, *331*, and *843*) did not produce a single, reproducible positive PCR band in any of the 18 populations at even this higher 2000c input level. Assuming the 2000c input level is a sufficient amount of cDNA to detect an OR expressed in merely 1 in ~2000 cells as estimated (~0.05% frequency in the population), and likely below the median frequency modeled by a normal distribution in the null hypothesis, the probability of observing “below average” expression in 18/18 samples would be <(0.5)^18^ = <10^−5^ (<1 occurrence in ~10,000 opportunities). Therefore, the observation that at least one of these 21 ORs (*Olfr843*, with no evidence whatsoever of near-threshold expression), and perhaps as many as 5 of the panel of 21 ORs exhibiting consistently below- or near-threshold expression in ~every sample, is an extremely unlikely outcome not predicted by the null hypothesis.

We note that the *p-values* used in the previous sections to reject the “null hypothesis” that OR representation is random from population to population (and OR subsets are instead significantly over-represented/under-represented) assume our nested PCR strategy is sensitive enough to monitor the upper part of a putative normal distribution (i.e., able to detect “above average”/”below average” levels of representation). The reasoning used to assert this depends on a very approximate estimation of OR transcript abundance per cell (see Methods). However, we note that the consequence of error in either direction does not significantly impact our overall conclusions, rather, merely shifts the claim towards more surprising OR over-representation (if our sensitivity is lower than estimated) or under-representation (if our sensitivity is higher than estimated).

### RNA FISH agreement with nested PCR

**Fig 3:**
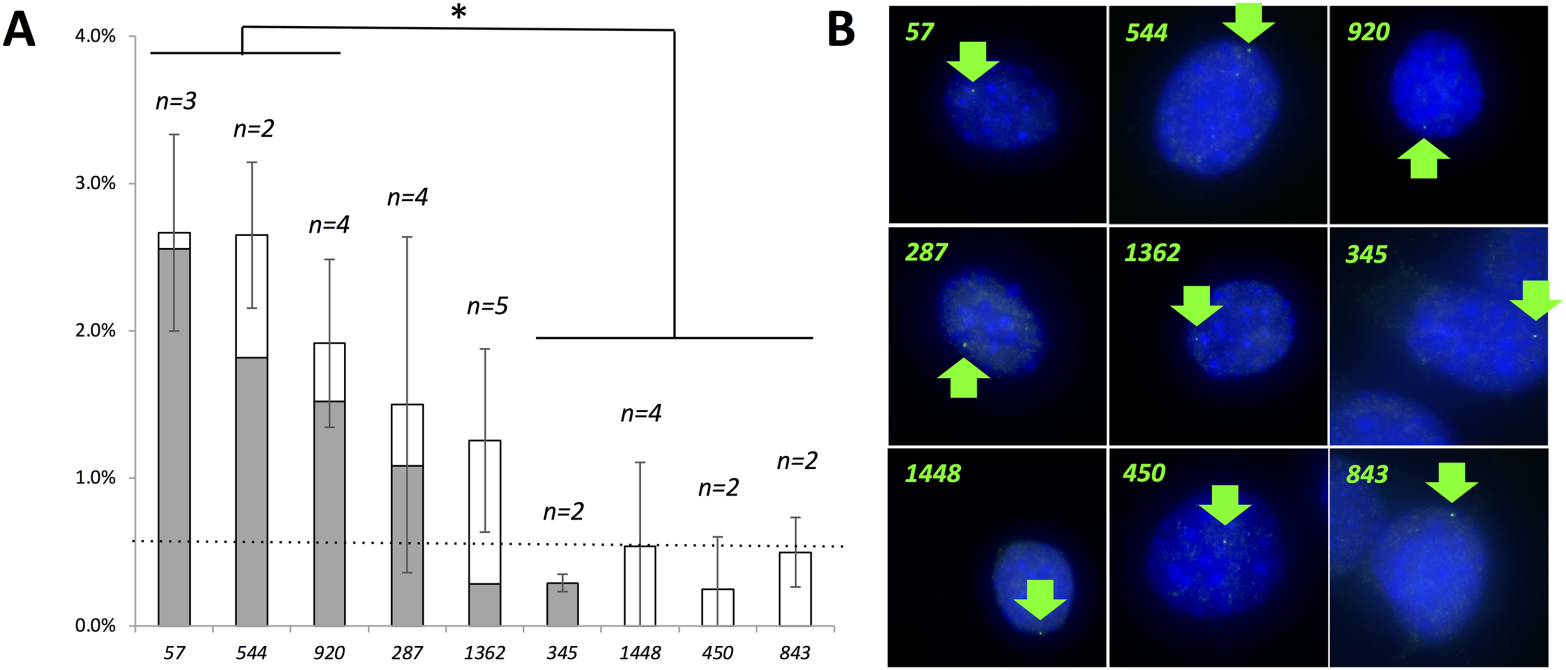
RNA FISH for 9 ORs conducted on replica *P13-Lineage-A* populations. Frozen cell stocks from *P11-Lineage-A* were grown in parallel to P13, and fixed for RNA FISH. Sense and antisense probes were produced for 9 ORs, including 3 robust expressers in this lineage (*Olfr920*, *Olfr544*, *Olfr57*), 2 medium expressers (*Olfr1362*, *287*), and 4 low/non-expressers (*Olfr450*, *345*, *1448*, *843*), as determined by nested PCR experiments (see Fig 2). **A**. Histograms report average positive frequencies in the P13 populations in replica experiments (number of replicas, *n*, is indicated above each bar), with error bars indicating one standard deviation. Sense probes were used to measure background false-positive noise; the average level of sense probe noise is indicated by the dotted line (0.6%). The gray portions of each bar represent “net positive frequency”, where sense probe noise is subtracted from antisense probe signal for each gene. T-test p-values are <0.5 for pairwise comparisons between the 3 robust expressers and 4 low/non-expressers (asterisk). **B**. Representative images showing one RNA FISH positive cell nucleus (signal indicated by arrows) for each OR probe (*Olfr* numbers indicated per panel).

We conducted parallel and blind RNA FISH experiments for three ORs identified in the lineage study as “robust expressers” (*Olfr920*, *544*, and *57*), two ORs identified as “medium” or “variable expressers” (*Olfr287*, *1362*), and four ORs identified as “low” or “non-expressers (*Olfr345*, *1448*, *450*, *843*) on multiple P13 cultures of *Lineage-A* to independently validate nested PCR results. The three robust expressers along these lineages (*Olfr920*, *544*, and *57*) also exhibited high positive frequencies as measured by these parallel RNA FISH experiments (>2% incidence), whereas two medium/more variable expressers along these lineages (*Olfr287*, *1362*) showed more modest positive frequencies (1–2%), as well as greater variability between samples (larger error bars) as measured by RNA FISH (Fig 3). In contrast, the four “low”/”non-expressers” (*Olfr450*, *345*, *1448*, *and 843*) were confirmed by RNA FISH as being at or below background noise levels as determined by sense probe controls (Fig 3). Therefore, two independent methods (RNA FISH and nested PCR) mutually reinforce the conclusion that a subset of robust OR expressers and low/non-expressers persists in OP6 cell populations.

### Bimodal distribution of OR expression probabilities

**Fig 4:**
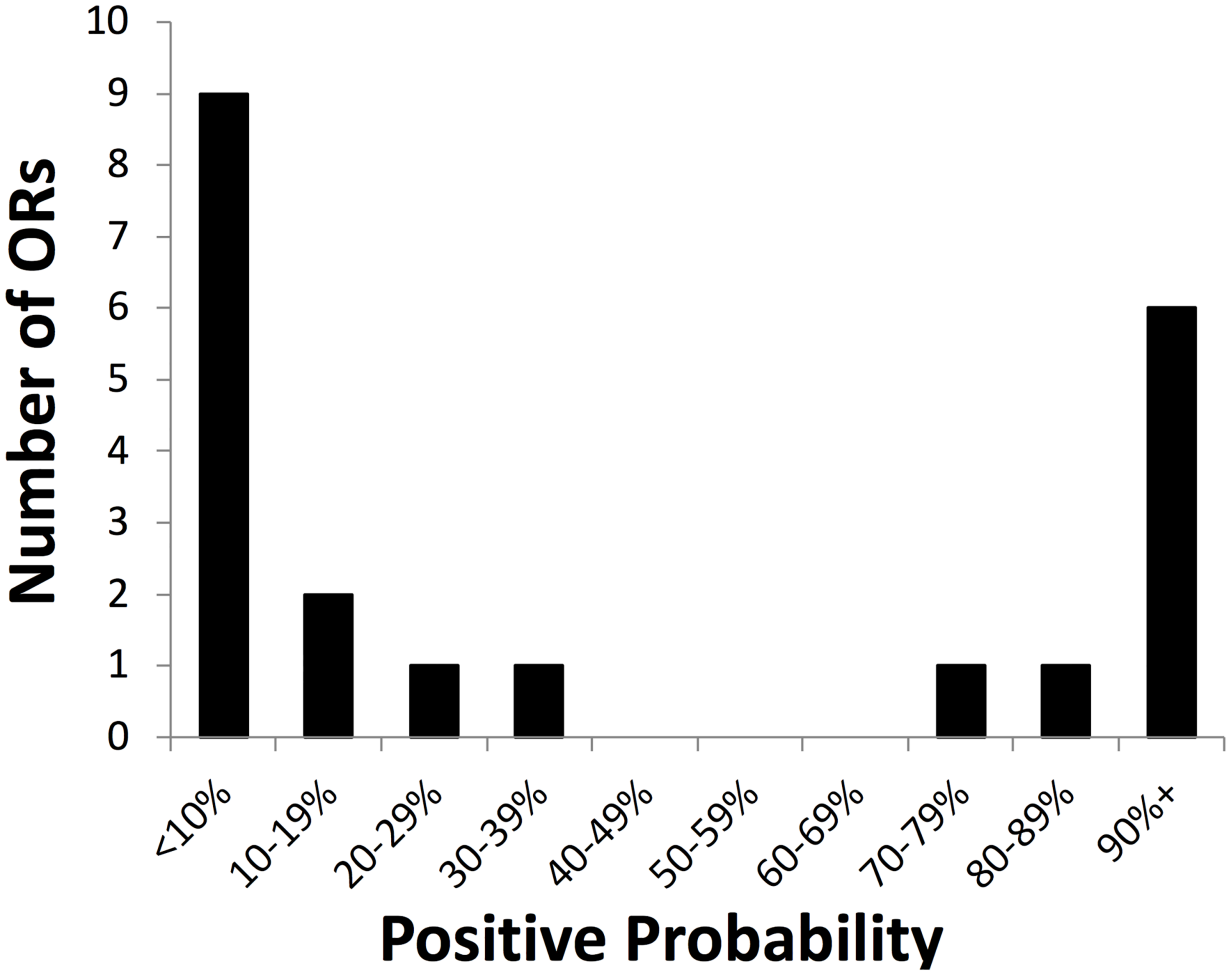
Bimodal distribution of nested PCR-positive probabilities. The 21 OR genes tested in this study are binned according to the probability of observing a PCR product at the 400-cell input level across 22 OP6 cultures in two divergent lineages (see Fig 3). This histogram illustrates a bimodal distribution of these positive probabilities.

Together, our data are most consistent with a bimodal distribution driven by strong selection biases that persists over multiple generations in these OP6 lineages (Fig 4). This bimodality is exhibited by an OR subset (~8 ORs) at one extreme that were consistently above-threshold in all 22 samples (with >70% detection probability even at the lower 400c input level), three of which whose high selection frequencies were confirmed by parallel RNA FISH experiments (Fig 3). At the other extreme we observed a subset of 9 ORs that were below-threshold in >90% of samples at the 400-cell input level (Fig 4), 5 of which were never reproducibly above-threshold even at the higher 2000c input level (Fig 2), and 4 of which were at or below background noise levels as evident by RNA FISH experiments (Fig 3).

While we observe these persistent global trends that suggest heritable OR selection probabilities, at higher resolution we note evidence for stochastic contributions that produce several unpredictable outcomes. For example, we observed consistently robust expression of *Olfr70* in *Lineages-A* and -*B* (72% detection probability at the lower 400c input level), yet this OR was consistently negative in *Lineage-C*. We also observed abrupt fluctuations evident along a lineage where some, mostly low-expressing/non-expressing ORs temporarily emerge as robust expressers for a few generations (e.g., in *Lineage-A*, the emergence of *Olfr1046* between P10-P13 or *Olfr307* between P13-16 at the 2000c input level). Similarly, our RNA FISH experiments gave quite variable frequencies in five different P13 cultures for *Olfr1362* and (especially) *Olfr287* (Fig 3). These inconsistencies indicate that OR demographics can vary significantly from one population to another. Therefore, we conclude that OR selection in OP6 cells is biased by a heritable epigenetic state that influences, but does not determine OR representation, resulting in sustained and largely predictable trends over long periods of culturing, against a backdrop of stochastic behavior.

## Discussion

For several years, we and others have used the OP6 cell line as a model for studying OR gene regulation. Based on previous work [30, 31, 33], individual OP6 cells appear to express only one dominant OR allele (monogenic, monoallelic), like mature OSNs *in vivo*. Therefore, the OP6 cell line provides an opportunity to investigate mechanisms of OR gene selection in a developmentally ~homogeneous (i.e., clonal) cell population. In this study, we have also taken advantage of the fact that OP6 cells have been immortalized, providing an opportunity to investigate the stability of OR choice, as well as the stability of OR selection tendencies, through cell division events.

We show that OR choice was generally not stable from one generation to the next. We have referred to this phenomenon as “OR re-selection” to potentially distinguish it from “OR switching”, a term used for a well-described phenomenon *in vivo* that prevents selection of OR pseudogenes [27, 28]. It is possible that the molecular mechanisms underlying “OR re-selection” and “OR switching” share some common features, but perhaps unlikely given the latter involves an active feedback loop whereas the former is presumably a passive event arising from chromatin re-organization through the cell cycle. “Re-selection” appears to be a common, if not obligatory, event during OP6 cell culturing. We note that from a total of 43 small/large colonies in which there was an opportunity to observe maintenance of OR selection for longer than merely a single generation, we identified only one case in which the same OR gene might have persisted in sibling cells (S1 Fig, Panel C, right panel in which *Olfr58* signal is evident in two nearby cells).

Two epigenetic events are associated with OR choice *in vivo*: the selected OR gene uniquely exhibits H3K9 demethylation and H3K4 methylation. Given these are presumed to be heritable (i.e., “epigenetic”) marks, the lack of OR selection memory in OP6 cells could indicate that these local chromatin changes have not occurred, raising the possibility that these marks accumulate as a consequence of expression and/or at a later commitment stage, as opposed to being causative/instructive with respect to initial OR selection. Alternatively, if these marks have occurred concurrently with OR selection and initial expression in OP6 cells, then these marks might not be robustly maintained during chromatin reorganization during the cell cycle.

Although OR choice itself does not appear to be heritable, we show evidence that OR selection probability is at least a partially heritable property, albeit not in an overly deterministic way. Our RNA FISH and nested PCR data are consistent with a model in which subsets of OR genes retain a selective advantage over others for prolonged periods of passaging, presumably influencing the probability of selection against a backdrop of stochastic drift.

These observed biases might hypothetically be explained by some form of persistent epigenetic memory that might have been established in the OP6 founder cell prior to isolating it from its developmental niche. For example, mature OSNs from late- or post-developmental mice exhibit an OR selection bias in the context of dorsal-ventral patterning in the olfactory epithelium, whereby ORs are eligible for selection only in a confined geographic zone [8]. This restricted eligibility is thought to be orchestrated by environmental cues, as opposed to being intrinsically determined by geographically localized stem cells that ultimately give rise to neurons in a specific region of the epithelium (Coleman *et al*., submitted; unpublished data, Schwob and Lane labs). We note that the specific subset of ORs that appear to be robustly represented in OP6 populations do not correlate with the spatial patterns established across the dorsal-ventral axis of adult mice (i.e., we observe OR expression from multiple of the canonical dorsal-ventral “zones”). This is perhaps not surprising given that OP6 cultures have been isolated away from putative developmental cues and given that the E10 olfactory placode from which the OP6 founder cell was derived has not yet developed the dorsal-ventral infrastructure that might generate such cues [21].

Alternatively, little is known about OR selection biases within the nascent E10 placode–for example, it might be important to express a subset of OR genes that facilitate the proper assembly of the earliest pioneering OSNs that are the first to navigate to glomeruli targets of the brain. A previous study characterized a small set (<20) OR genes that first appear at early mouse embryonic stages (E10-E13) [22], however there does not appear to be enrichment of these specific early OSN expressers in OP6 populations.

Instead, we consider the possibility that OP6 populations now removed from a developmental context for several generations might exhibit OR selection biases that reflect intrinsic, as opposed to extrinsic influences. This hypothesis raises interesting questions about the putative epigenetic mechanism underlying the observed heritable OR selection trends, perhaps reflecting an intrinsic default state for naïve OR loci in the absence of specification/developmental cues. Elucidating the molecular mechanisms that maintain these biases over many cell divisions in a non-developmental context could provide insights into OR regulation *in vivo*. For example, if these biases reflect different native activity levels of local enhancers and/or different local chromatin states, then we might eventually clarify a role for these features in the competitive *trans* selection/refinement process that transpires *in vivo* [43, 44]. Alternatively, a deeper understanding of these observed biases might elucidate native organization of local chromatin that facilitate increased access to a putative expression hub, such as the topologically associating domains (TADs) that have been proposed to facilitate OR expression in the absence of local enhancers [10, 45]. Future studies in the OP6 cell line aim to characterize chromatin states and local enhancer activities in order to better understand why certain OR genes/loci consistently outcompete others in this system.

## Supporting information

S1 Fig RNA FISH images for all positive cells identified within small and large OP6 colonies. **A**. Arrows indicate positive RNA FISH signals for the one positive cell identified within 15 small OP6 colonies for a panel of probes (*Olfr* number is indicated to the left of each panel). **B**. Arrows indicate positive RNA FISH signals for the one positive cell identified within 19 large OP6 colonies for a panel of probes (*Olfr* number is indicated to the left of each panel). In each case, only a portion of the large colony containing the positive cell is imaged. **C**. Arrows indicate positive RNA FISH signals for the two positive cells identified within 9 large OP6 colonies (*Olfr* number is indicated to the left of each panel). In each case, only a portion of the large colony containing the positive cell is imaged. In most cases, the two separated positive cells were captured in two separate images (dotted lines delineate two separate images taken of different regions of the same colony).
(PDF)

S2 Fig Representative gel images for nested PCR conducted on 100 gDNA templates (*g*), RT (*c*), and no-RT (-), for replica experiments (*R1*, *R2*) at two cDNA input levels (*400*- and *2000*-cell) on *P13a*/*Lineage-A* for the 21 OR genes used in the lineage study. The *Olfr* number is indicated to the left of each panel, enclosed within a shaded box depicting robustness of PCR products relative to gDNA controls at the *400*-cell input level (see Fig 2).
(PDF)

S3 Fig Nested PCR results for the panel of 21 OR genes (see Fig 2) on an independent OP6 lineage (*Lineage-C*), analyzing populations harvested at P8, P9, P9, P12, P13, and P13. Robust positives (>100 templates) are shaded dark gray, faint positives (<100 templates) are shaded light gray, negatives are unshaded.
(PDF)

S1 Table All oligonucleotide primer sequences used in nested PCR assays, as well as for production of RNA FISH probes, are provided. (PDF)
