# Supplementary figures and images for: Frequent and biased odorant receptor (OR) re-selection in an olfactory placode-derived cell line
Source: PLoS One. 2018 Sep 26;13(9):e0204604. doi: 10.1371/journal.pone.0204604 (PMC6157871; doi:10.1371/journal.pone.0204604)

**A.**

**58**

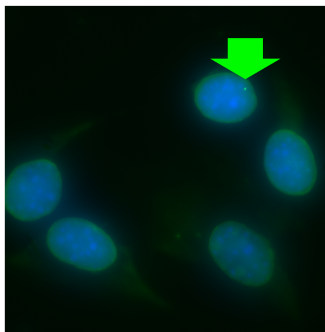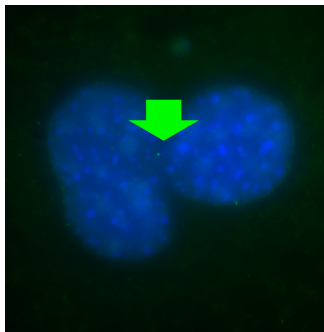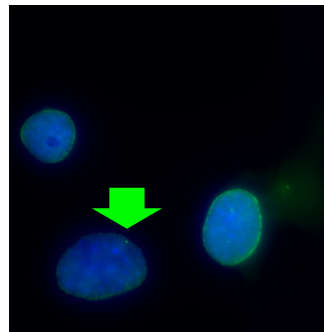

**378**

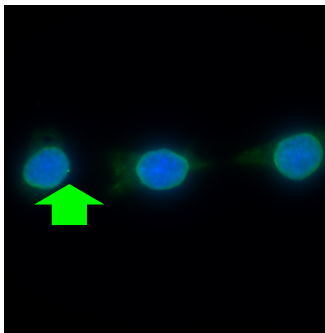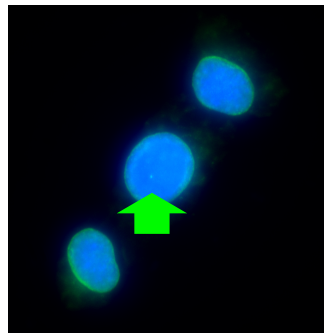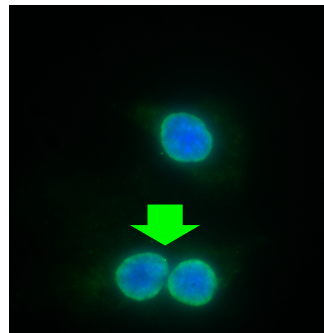

**287**

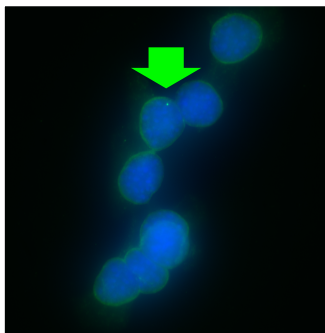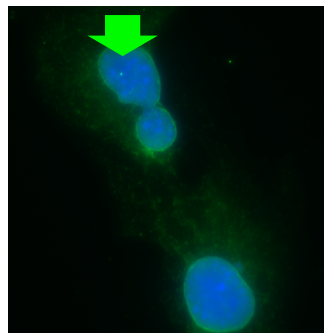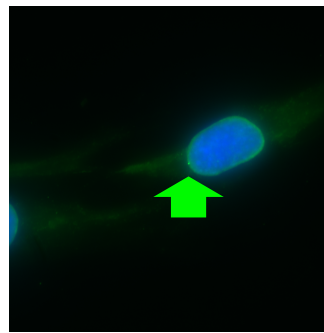

**860**

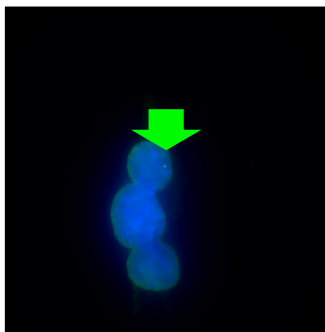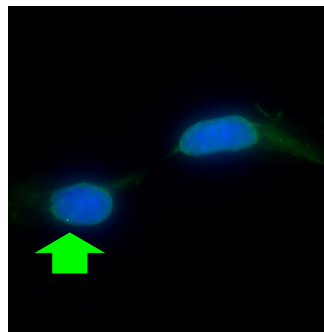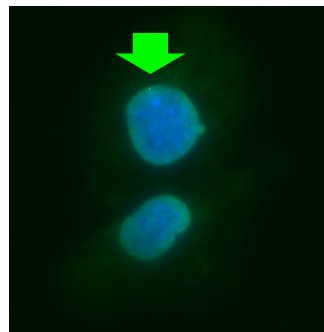

**69**

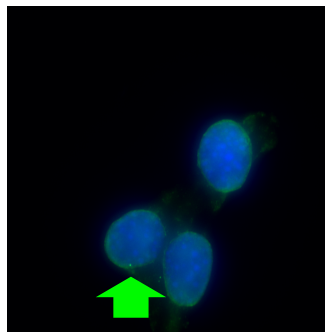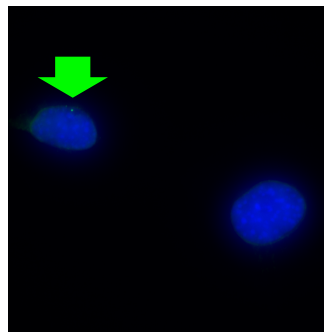

**868**

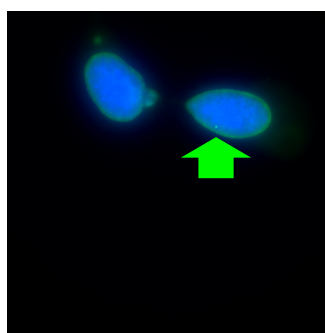

**B.**

**58**

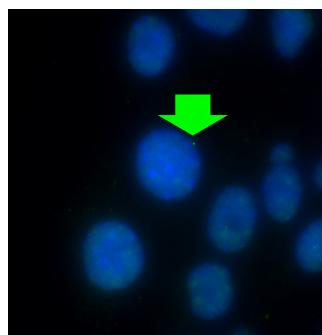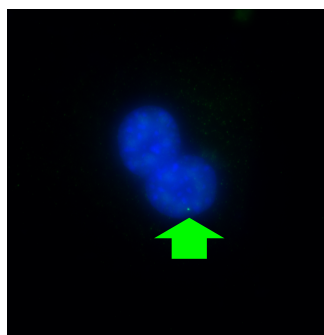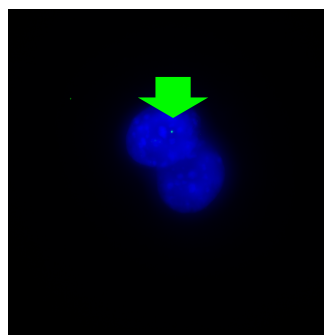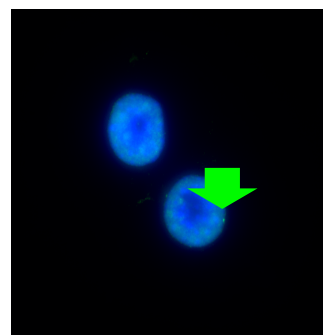

**378**

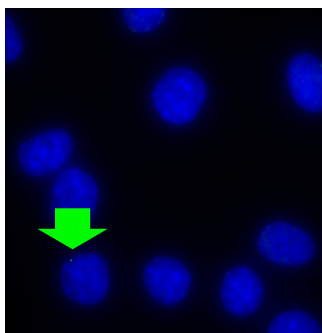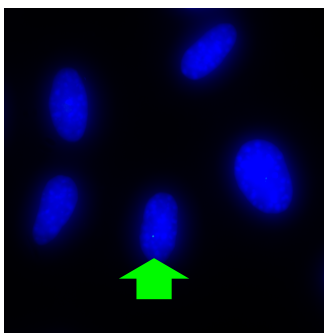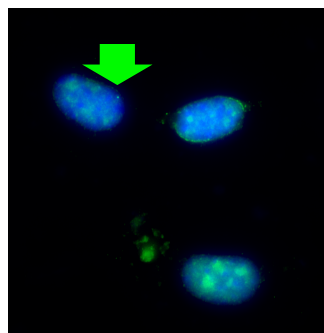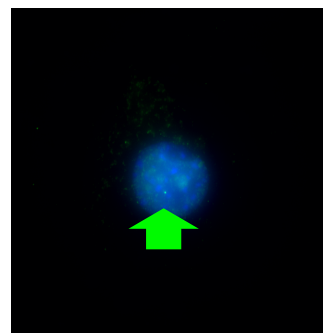

**868**

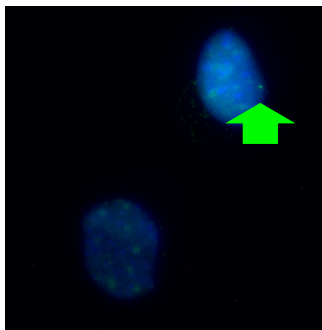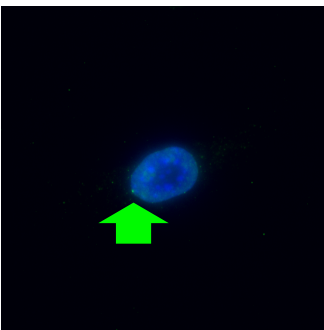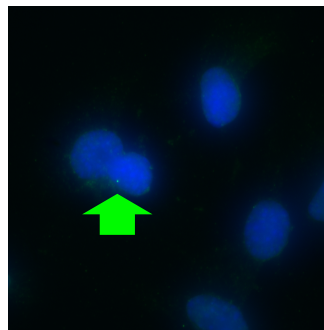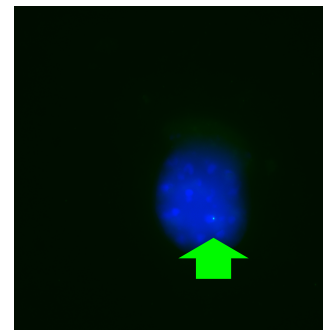

**860**

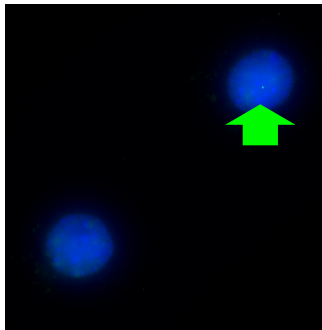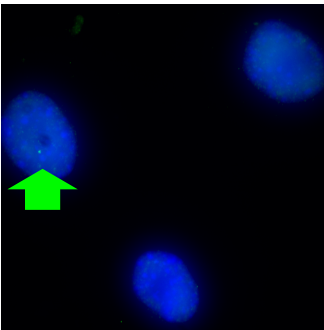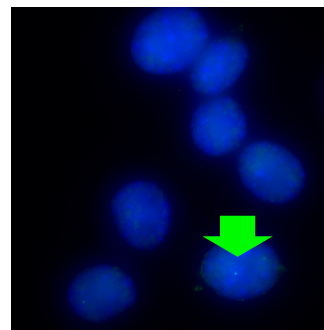

**287**

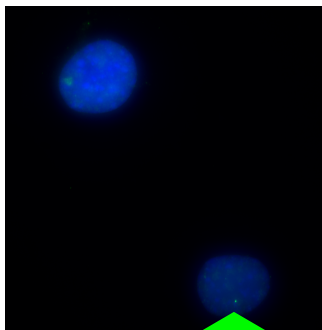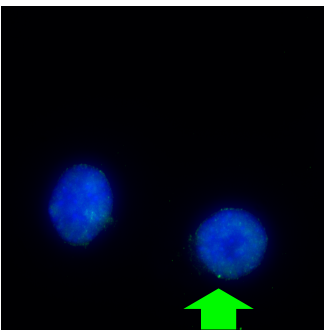

**69**

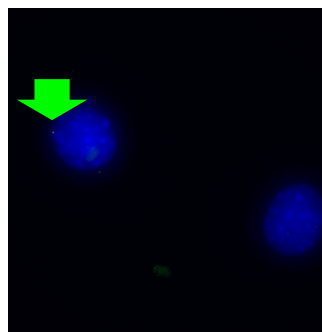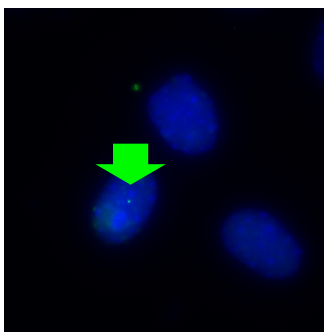

**C.**

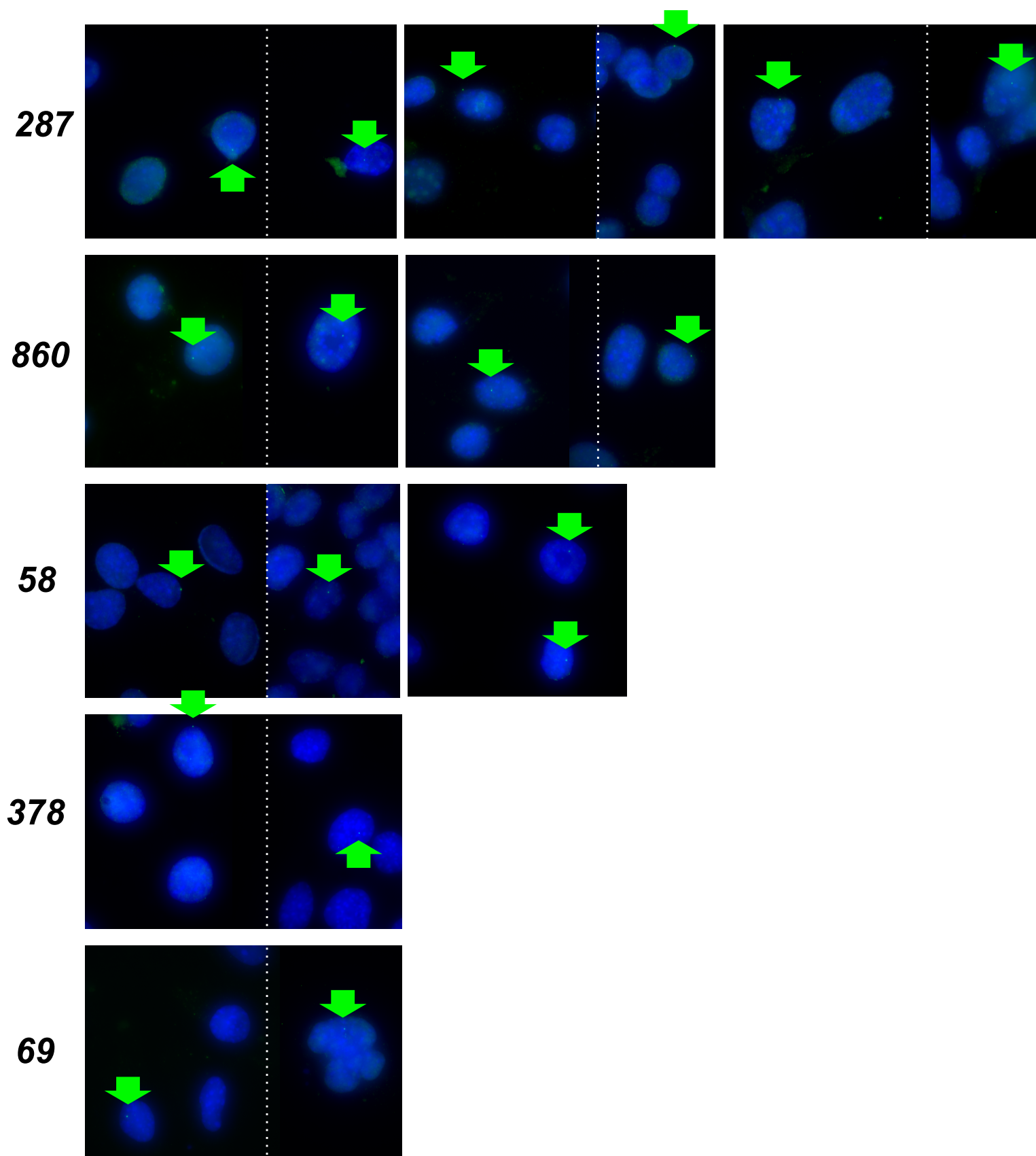

Supplement: S1 Fig — A. Arrows indicate positive RNA FISH signals for the one positive cell identified within 15 small OP6 colonies for a panel of probes (Olfr number is indicated to the left of each panel). B. Arrows indicate positive RNA FISH signals for the one positive cell identified within 19 large OP6 colonies for a panel of probes (Olfr number is indicated to the left of each panel). In each case, only a portion of the large colony containing the positive cell is imaged. C. Arrows indicate positive RNA FISH signals for the two positive cells identified within 9 large OP6 colonies (Olfr number is indicated to the left of each panel). In each case, only a portion of the large colony containing the positive cell is imaged. In most cases, the two separated positive cells were captured in two separate images (dotted lines delineate two separate images taken of different regions of the same colony). (PDF) [file pone.0204604.s001.pdf]

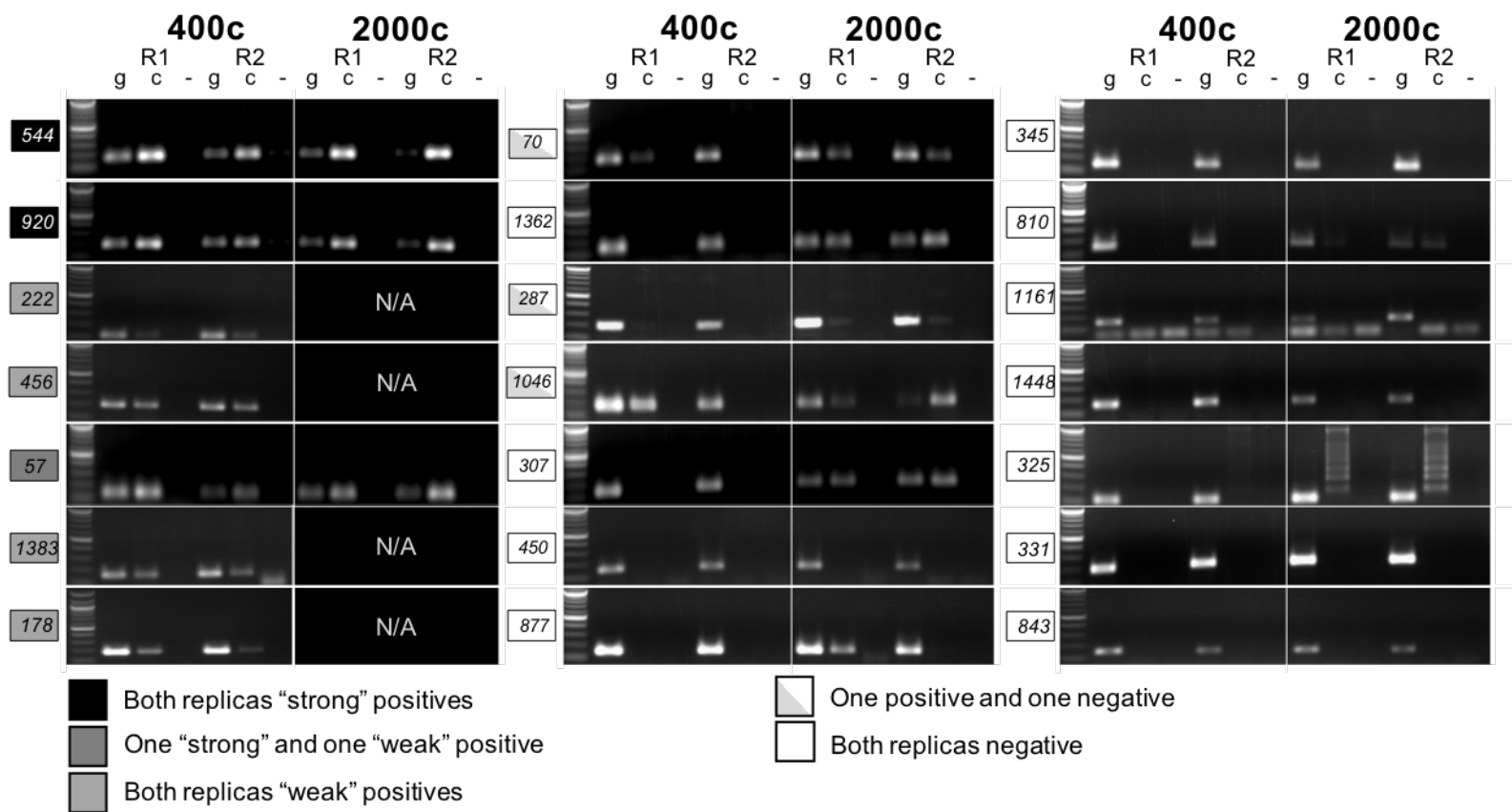

Supplement: S2 Fig — The Olfr number is indicated to the left of each panel, enclosed within a shaded box depicting robustness of PCR products relative to gDNA controls at the 400-cell input level (see Fig 2). (PDF) [file pone.0204604.s002.pdf]
