# Supplementary material for: Frequent and biased odorant receptor (OR) re-selection in an olfactory placode-derived cell line
Source: PLoS One. 2018 Sep 26;13(9):e0204604. doi: 10.1371/journal.pone.0204604 (PMC6157871; doi:10.1371/journal.pone.0204604)
Supplement: S3 Fig — Robust positives (>100 templates) are shaded dark gray, faint positives (<100 templates) are shaded light gray, negatives are unshaded. (PDF) [file pone.0204604.s003.pdf]

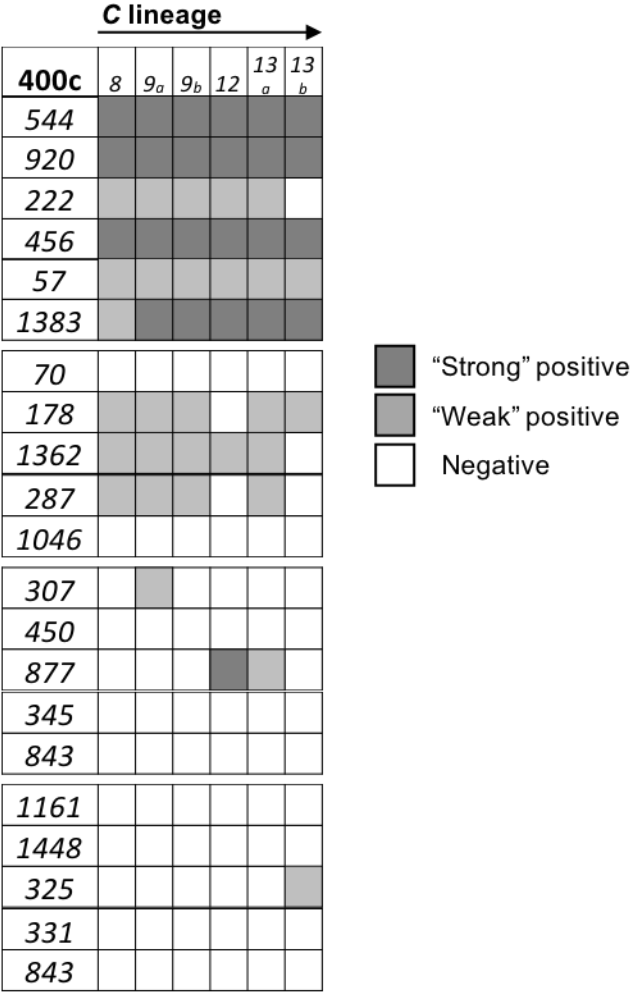
C lineage
400c
8
9a
9b
12
13 a
13 b
544
920
222
456
57
1383
70
178
1362
287
1046
307
450
877
345
843
1161
1448
325
331
843
"Strong" positive
"Weak" positive
Negative
