# Supplementary material for: Frequent and biased odorant receptor (OR) re-selection in an olfactory placode-derived cell line
Source: PLoS One. 2018 Sep 26;13(9):e0204604. doi: 10.1371/journal.pone.0204604 (PMC6157871; doi:10.1371/journal.pone.0204604)
Supplement: S1 Table — (PDF) [file pone.0204604.s004.pdf]

	Olfr	**Forward**	**Reverse**	**Size**
**Outer nest primers**				
**exon**	544	TCGCTTCTGTCGCTCAGATG	TGAAGACCTGTTGCTGAGTCC	449
	920	GTGCTGAATTCTCACCTTCATAC	AAGGGAGGAGATCACAGAAATAG	403
	222	AAGCTGTCCTGTGCTGATAC	GCTCCCTTCACGTCCTTATT	344
	456	TCTCTGGTCTCTCTGTCCTATG	GCATTGGTGTATGGGCTGTA	397
	57	CTTACTGCTTTTTTCAGGGTTA	TGTTGCAGTAGCATGTGAG	511
	1383	CTGGGTGGAACTGAGTGTATG	CACATGGACAGGTTGGAGATAG	474
	178	CACTCCCAAGATGCTAGAGAAC	CCAAGACAGTGGTAATGGTAAGA	416
	70	TGTGCTCTGTCTGGGTATCT	GCTGTGGCCACCGTTAATA	528
	1362	TCGCCTGGAGATGGTACTCT	ATGGCCAAAGTGAAGGACAA	549
	287	CTTTGGGATGGTCAATGGTATCT	TGGTGTAGAGGAGAGAGAAAGG	389
	1046	CTACACAGGGTGTGCTGTGC	AGCTGCACGTGGAGAAGG	449
	307	CCAATCCCAAAGTTCGTTG	AAACACCCATCATCAGCAC	214
	450	TTCCTTTTGGGAGCAATGTC	CAGTGAGGTGAGAGGCACAAG	400
	877	GGATTAACAGACCAGCCTCAA	CATCCCATAGACACCTGACAAA	408
	345	TTATGGCCACATTGGAGTCAC	GCTCCTTTCATATCCCGATTC	249
	810	GGTTTGACTGATGACCCACA	CCATTGTTTCCAGGAGCTGT	550
	1161	GGGCCATGTTCATCCTATTG	GCCACAAATCTGTCATAGGC	349
	1448	GCAGGATTAACAGATGACCCAAAG	GTCTCACTGATATAGGTGTCTGAGCA	548
	325	GCCTCCTGCTGATGTCTTTAT	CTTCATAGCCTCTGTGACATTCT	485
	331	CATACCAATCCACCTCCACATAC	GAGCACACAGCACACATACA	466
	843	CCTGTCCCAGGTGTGTTTTG	TGGAGATGATAAGACCACAGGA	649
**Inner nest primers**				
**exon**	544	GTCCTGGATATGCTCCTGTTAG	ACACGGTAGACAATGGATGAG	170
	920	CTGTGTCATTTTGGAGTGTTATATTCT	GCCATCACAGAACGACAGTCTCAGG	196
	222	TCCTCTCCTACGGCTTCAT	TAGAAGAGGGAGACCACCAA	112
	456	TCTCTGGTCTCTCTGTCCTATG	GAAGATCACTACCACCTTGTCC	198
	57	GGTGTTACGGCTGTCATTCT	GCCAGTAGCATAGCTGTAATGTA	135
	1383	ATGCATTGGCCATCTCCTC	TCTGTTCCTCCTGTGTCCT	160
	178	GCTGAAACTTCAGACTGCTTTC	GTTAACCTTAAGAGACACCCTACA	182
	70	CTTCATCAGCAACCTCTCTCTC	TATGCCATCACAGCCAGAAG	177
	1362	CATCCCACAGATGCTGGTAAA	GGCAGCATAGCGGTCATAA	145
	287	GTGCTCATCATCACCTCCTATG	AAACATGGCTGAGGTGTAGAA	138
	1046	CCATCTGCAAACCTCTTCTCT	CAGAGGGACACAGTCACAATAA	179
	307	TTCTATTTCCGTTCTGGCTTGT	TCATGATGGCACCATAGTTCAG	140
	450	GTTGCTTGATGGCTGGTTTC	ACCATGACCTTGTTGAAAGAGA	154
	877	GCTGAACTCTCACCTGCATATC	CTGTGTCATACACCCTGCATAG	151
	345	CCATTCTGAAGACTCCCTCTATC	GGCATCCTTGTCATTAGTGTTATT	146
	810	CTGGCAGCCATGTCCTATG	CTAACAGCAGTGGTGGAAAGA	145
	1161	CTTGGTGTTTCTCGCCATCTA	GGGAGCAATGACAGAGGAATAG	154
	1448	TTCTTCTTCTTCGTGGGCTTTA	ATTCAGGAAGCCACAGACATAG	165
	325	AGCAGAGGGCAGGAAGAA	GGAGGGAGGCAGGGAAA	107
	331	GCCATGTCCTATGACCACTATG	GGACCTCACAGAAGAAGTGATG	196
	843	GTCTCTGTGTACTGCTCGTTT	GCAGGCATGTTTGATGACTTG	152

	Olfr	Forward	Reverse	Size
**RNA FISH**				
**Intronic probes**	58	TCAAAGACAAAATACATCATTAAGCAAAG	ACCATTGTAGAGAACTAAGCAGTATAG	2550
	287	CACTACTGCCTAGTCTTTATTATTATTATT	CCACAATTCGGTTCCCATTC	3043
	860	AGAGATGAATCAGTGGTGAGATG	GGTGATGAACTAAGCACTACAGA	2493
	378	TTCCTGTCCTTCAAGCTTGAG	AGCAGCAGTTAACATTCCCA	2317
	69	GATTAAAGCAGTATTTTTGAAAACCAGA	TTCACCAGAGTGGGAGGATA	2561
	868	TATCAAAATTGGTAATCTATTAAAGAGTAT	AGGTTTCCCACATAACTACAG	1553
	544	CCTCCAGTCTGATGCTCTTTC	CGCTTGAGTAGTCTTGCTATG	1406
	843	ATTCAGGGTTCTCCACGTAAAT	TCCTCCAGTCCTCATATCTCTT	1105
	57	CCGGCTATCTCCTTCATTT	GAGGCTGGAACATGGTTAAT	1400
	1364	GGGAGCCTTAAGAACTAACGAA	GGGAGGAGCCAAATGAGAAA	1431
	450	CAGGACTCTCTGCAAGTCATC	GCAAGCTGACACTTCTGAATTT	1409
	1448	TGTAGCAGAAGATGGCCTAGT	GCTGCCCTGTATCAAAGAGAAA	1427
	345	GGGTGGTGGTATGATTCTTGA	CTGTGAGTTAAAGAGCAAATGGG	1471
	287	CACTACTGCCTAGTCTTTATTATTATTATT	CCACAATTCGGTTCCCATTC	3043
	920	GTCAGTAACATAGTTGTGCTC	CTAAAACAAAGCACATTTGGAGG	2108
